# Benefit of Preoperative Flexible Endoscopy for Patients Undergoing Weight-Reduction Surgery in Saudi Arabia

**DOI:** 10.4103/1319-3767.37795

**Published:** 2008-01

**Authors:** Ahmad M. Al Akwaa, Ahmad Alsalman

**Affiliations:** Department of Medicine, King Abdulaziz Medical City, Alahsa, Saudi Arabia

**Keywords:** Bariatric surgery, flexible endoscopy, morbid obesity, preoperative evaluation

## Abstract

**Background:**

Little information is available to demonstrate the importance of flexible endoscopic examination of the upper gastrointestinal tract in obese patients prior to the weight-reduction surgery. In spite of the controversies, there are more evidences to support the value of preoperative endoscopy. In this study, we aimed to evaluate the benefit of preoperative endoscopy in morbidly obese patients who have planned to undergo bariatric surgery.

**Materials and Methods:**

The medical records of morbidly obese patients who were admitted to our hospital from November 2004 to January 2007 and underwent flexible esophagogastroduodenoscopy (EGD) prior to the weight-reduction surgery were reviewed. The endoscopic findings and demographic data were recorded and analyzed.

**Results:**

Sixty-five patients underwent EGD preoperatively. The mean age was 34.6 years (range: 18–52 years), their mean BMI was 57 (range: 35–92) with a maximum weight of 280 kg. Majority were females (64%). Endoscopic findings included gastritis in 44 patients (67.7%), hiatus hernia in 8 (12%), gastric erosions in 7 (10.7%), reflux esophagitis in 4 (6%) and normal EGD findings in 15 patients (23%). There was no significant increase in reflux esophagitis in this group of patients. Sixty percent of the patients had comorbid medical conditions with diabetes mellitus being the most common.

**Conclusion:**

These data suggest that it might be necessary to perform preoperative EGD in patients undergoing bariatric surgery, although it possibly will not alter the surgical intervention. Prospectively conducted studies with larger number of patients are required to further explore the need of EGD in this subset of patients.

Esophagogastroduodenoscopy (EGD) is a very important diagnostic and therapeutic tool for upper gastrointestinal disorders.[1,2] Although it is invasive, yet it is a safe and effective procedure.[1,2] Routine EGD has been utilized as part of the preoperative workup in morbidly obese patients who have planned to undergo bariatric surgery.[3–5] There are limited and inconsistent data on the value of preoperative EGD in morbidly obese patients undergoing weight reduction surgery.[3–6] Similarly, there is no available data on this subject from Saudi Arabia. Endoscopic pathological findings may have influence on the management of these patients.[3–5] It is unclear whether to subject these patients to EGD before surgery or not.

In this study we aimed to find out the importance and benefits of preoperative EGD in morbidly obese patients planned for weight reduction surgery.

## MATERIALS AND METHODS

The medical charts and endoscopy reports of the patient who were referred for EGD prior to bariatric surgery in the period between November 2004 and Jan 2007 were reviewed. Demographic data, comorbid conditions, any gastrointestinal symptoms, medications and other complications were recorded and tabulated.

## RESULTS

Sixty-five patients underwent preoperative endoscopic evaluation. Their mean age 34.6 (range: 18–52 years) and mean BMI 57 (range: 45–92). There were 42 females and 23 males. Demographic data is shown in Table 1.

**Table 1:** Demographic data of 65 patients

Age	Mean: 34.6	Range: 18-52 years
Sex	F = 42, M = 23	
BMI	Mean: 57	Range: 35-92
Weight	Mean: 145 kg	Range: 96-280 kg

**Figure 1:**
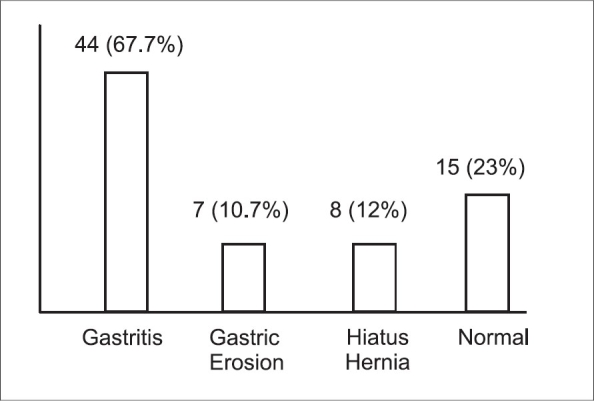
Endoscopic findings in 65 morbidly obese patients

The main endoscopic findings were gastritis in 67.7%, followed by hiatus hernia in 12% and then gastric erosions in 10.7% of patients. Normal endoscopic findings were observed in 23% of patients [Figure 1]. Other findings are shown in Table 2. Infrequent findings included large gastric polyps ∼3 cm which was removed endoscopically by using the polypectomy snare - the histology revealed hyperplastic polyp with hamartomatous picture and no evidences of malignancy. A large gastric antrum mucosal fold was encountered in one patient. Bile reflux was found in 2 patients. Comorbid conditions (mainly diabetes mellitus and Hypertension) were reported in 39 (60%) patients; most of them were on multiple medications [Table 3]. Twelve patients administered low-dose aspirin. There were no significant differences in the endoscopic findings between the patients with and without comorbid conditions, except the gastric erosions were more in patients with comorbid conditions [Table 4].

**Table 2:** Infrequent endoscopic findings

Duodenal Erosions	3
Duodenitis	3
Deformed duodenal bulb	2
Duodenogastric bile reflux	2
Large gastric polyp	1
Large antral mucosal fold	1
Reflux esophagitis	4

**Table 3:** Comorbid conditions in 65 patients

DM	25
Hypertension	22
Bronchial asthma	5
Hyperlipidemia	5
Hypothyroidism	4
Obstructive sleep apnea	4

**Table 4:** Endoscopic findings in relation to comorbidity

	Absence of comorbid conditions	Comorbid conditions
Number	26	39
Normal	5	10
Gastritis	18	24
Reflux esophagitis	2	2
Gastric erosions	2	6
Duodenal erosions	2	1

Seventy-five percent of patients had either gastroduodenitis or erosions. Gastrointestinal symptoms were retrieved from nine charts; half of them had normal endoscopic findings. All the four patients with reflux esophagitis had BMI > 70.

## DISCUSSION

Flexible endoscopy is an important investigative tool for upper gastrointestinal disorders (GIT).[1,2] It has been shown to be essential in preoperative workup for patients planned for bariatric surgery.[3,4] Our study sample is small due to the lack of popularity of such surgeries in weight reduction; in addition to that, all the patients did not undergo EGD preoperatively. Recently, there has been a growing acceptance and public awareness on such surgeries as an effective means of weight reduction. Endoscopic gastritis was very common (67.7%) in comparison to other studies; this could be attributed to high prevalence of helicobacter pylori infection in our area.[7,8] Zeni *et al.* reported gastritis in 27% of patients undergoing EGD for preoperative evaluation of laparoscopic Roux-en-Y gastric bypass.[3] Unexpectedly, no gastric or duodenal ulcer was observed in this study, which can be explained on the basis of the fact that the EGD was done as part of preoperative evaluation, and not specifically for the investigation of symptomatic patients.[2] The occurrences of gastric and duodenal erosions were similar to what is reported in the literature. The correlation between morbid obesity and increase in the incidence of reflux esophagitis has been shown by several recent studies in a range of 17–33.3%.[3,9–12] We failed to show this correlation; however, our patients with reflux esophagitis had BMI > 70, which might demonstrate that the higher the BMI, the greater the chance of having reflux esophagitis.[9,11] The upper GIT symptoms have been shown to be predictive for the presence of pathological endoscopic finding, which might be the case in our study, although the number of our patients with reported symptoms is small.[6] Moreover, our review of patient-charts did not reveal whether these patients were actually symptomatic.

Patients with comorbid conditions taking multiple medications, including aspirin, had almost similar findings to those with no comorbid medical problems except for gastric erosions, which were higher in patients with comorbid conditions. The high percentage of hiatus hernia (17–52%) reported in obese patients was not noticed in our patients.[3,9,11]

Endoscopic findings such as hiatus herniae, and benign or malignant gastric tumors have altered surgical intervention in some reports.[3,5] However, in our study, scheduled surgeries remained unchanged since none of the endoscopic findings were serious enough to mandate rescheduling of surgery. The study findings were not major and did not lead to changes in the surgical plans; therefore, it might not be wise to expose these patients routinely to an invasive procedure, which is relatively costly and uncomfortable, with a potential risk although it is minimal. In this study, the number of patients is small to draw a definite conclusion, but it could serve as a baseline for further studies to be conducted in a prospective manner.

## CONCLUSION

In morbidly obese patients, the prevalence of upper EGD pathological findings is significant, although they might not alter the future surgery plans. Further prospective studies with larger cohorts are required to evaluate the need for flexible endoscopy in obese patients opting for bariatric surgery.

## References

[1] Blades EW, Chak A, Sivak MV (1994). Uper gastrointestinal endoscopy. Gastrointestinal Endoscopy Clinic of North America.

[2] Ayoola EA, al-Rashed RS, al-Mofleh IA, al-Faleh FZ, Laajam M (1996). Diagnostic yield of upper gastrointestinal endoscopy in relation to age and gender: A study of 10112 Saudi patients. Hepatogastroenterology.

[3] Zeni TM, Frantzides CT, Mahr C, Denham EW, Meiselman M, Goldberg MJ (2006). Value of preoperative upper endoscopy in patients undergoing laparoscopic gastric bypass. Obes Surg.

[4] Sharaf RN, Weinshel EH, Bini EJ, Rosenberg J, Sherman A, Ren CJ (2004). Endoscopy plays an important preoperative role in bariatric surgery. Obes Surg.

[5] Schirmer B, Erenoglu C, Miller A (2002). Flexible endoscopy in the management of patients undergoing Roux-en-Y gastric bypass. Obes Surg.

[6] Korenkov M, Sauerland S, Shah S, Junginger T (2006). Is routine preoperative upper endoscopy in gastric banding patients really necessary?. Obes Surg.

[7] Al-Quorain A, Satti MB, Al-Hamdan A, Al-Gassb G, Al-Freihi H, Al-Gindan Y (1991). Pattern of upper gastrointestinal disease in the eastern province of Saudi Arabia. Endoscopic evaluation of 2982 patients. Trop Geogr Med.

[8] Rashid RS, Ayoola EA, Mofleh IA, Chowdhury MN, Mahmood Kfaleh FZ (1992). *Helicobacter pylori* and dyspepsia in an Arab population. Trop Geogr Med.

[9] Wilson LJ, Ma W, Hirshowitz BI (1999). Association of obesity with hiatal hernia and esophagitis. Am J Gastroenterol.

[10] van Oijen MG, Josemanders DF, Laheij RJ, Van Rossum LG, Tan AC, Jansen JB (2006). Gasrointestinal disorders and symptoms: does body mass index matter?. Neth J Med.

[11] Suter M, Dorta G, Giusti V, Calmes JM (2004). Gastro-esophageal reflux and esophageal motility disorders in morbidly obese patients. Obes Surg.

[12] El-Serag HB, Graham DY, Satia JA, Rabeneck L (2005). Obesity is an independent risk factor for GERD symptoms and erosive esophagitis. Am J Gastroenterol.

